# Early-onset breast cancer in a Lebanese family with Lynch syndrome due to MSH2 gene mutation

**DOI:** 10.1186/1897-4287-7-10

**Published:** 2009-05-28

**Authors:** Riad Akoum, Albert Ghaoui, Emile Brihi, Maroun Ghabash, Nicolas Hajjar

**Affiliations:** 1 Department of Oncology, Rizk Hospital, Beirut, Lebanon; 2 Department of Medicine, Notre Dame du Rosaire Hospital, Beirut, Lebanon

## Abstract

**Background:**

There are still controversies about the integration of breast cancer as a part of the disease spectrum in Lynch syndrome.

**Methods:**

A regular follow-up of a Lebanese pedigree with Lynch syndrome due to a point mutation of MSH2 gene at the splice donor site of intron 3 started in 1996.

**Results:**

A 26-year-old pregnant woman, mutation carrier, developed an aggressive breast cancer, refractory to standard chemotherapy regimens. The microsatellite analysis of the tumor showed an unstable pattern for markers BAT25 and BAT26. The immunohistochemical staining was negative for MSH2 and MSH6 and normal for MLH1 and PMS6 enzymes.

**Conclusion:**

The segregation of the mutation with the disease phenotype and these results suggest that MSH2 inactivation may be involved in the accelerated breast carcinogenesis and might be considered in the cancer screening program.

## Background

**Figure 1:**
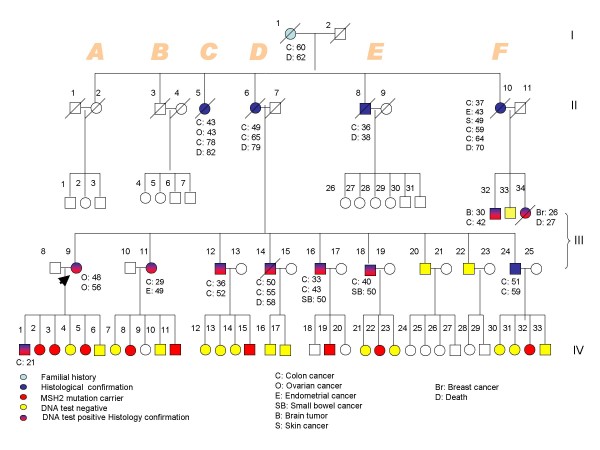
**Analysis of the Lebanese pedigree with Lynch syndrome due to MSH2 gene mutation**.

The identification of the germline mutation in a Lynch syndrome family allows mutation carriers to be included in lifesaving cancer surveillance programs [1]. The occurrence of cancer of a type that is atypical for the hereditary cancer syndrome in a family makes the interpretation of the pedigree difficult. It is not uncommon to see Lynch syndrome pedigrees with breast cancer (BC). However, there is no agreement as to whether breast cancer is part of the disease spectrum [2-4]. Most genetic and immunohistochemical studies on familial and sporadic breast cancers did not evoke any strong relationship with the mismatch repair (MMR) gene defect. Muller et al [3] found that synchronous and metachronous breast cancers from Lynch syndrome families usually arise sporadically because they display a stable micro satellite pattern and normal MMR protein expression. Wong et al [5] in an extensive screening study of 59 multiple-case BC families; did not identify any genetic abnormality that might implicate MSH2 as a BC susceptibility gene. Khilko et al [6] in an immunohistochemical staining of 211 BC specimens did not show any loss of MMR protein expression. Here, we report a Lebanese Lynch syndrome family (figure 1) with a case of early-onset breast cancer, in which the microsatellite instability (MSI) and the immunohistochemical (IHC) studies suggest strongly a relationship with the MSH2 gene defect.

## Methods

Seventeen carriers of MSH2 gene point mutation, IVS3+1G>A, were included in a regular surveillance program. An eight-year follow-up result of this family was published in a previous report [7]. The MSI analysis was performed after PCR amplification of tumor and normal surrounding borders DNA at eight loci containing mononucleotide and dinucleotide repeated sequences using fluorescent specific primers for each locus: BAT-25, BAT-26, D2S123, D17S250, RIITGFβ, NR21, NR22, and NR24. The PCR products were electrophoresed for three hours in an Applied Biosystems (ABI) Prism sequencer. Data were collected using the GeneScan program for fragment analysis and alterations in the micro satellites were detected by comparing normal tissue and tumor tissue DNA strands in neighboring lanes. The IHC staining was also performed in paraffin embedded tumor and normal tissues for MSH2, MLH1, MSH6, PMS2, CK7 and CK20. Testing for germline BRCA1, BRCA2 and TP53 mutations was not available in our clinic.

## Results and discussion

Although no new colorectal or gynecological tumor was screen-detected since the previous update on this pedigree [7] an unforeseen breast cancer in a 26 years old pregnant female (III 34) has occurred. No family history of breast cancer was noted in any branch of the family. No breast or ovarian cancer was known in any paternal family member of this patient. Screening for breast cancer was not part of our surveillance program, since there was no reason to suspect such a tumor as early as 26 years and during pregnancy. This patient who carries the MSH2 germline mutation developed an infiltrating ductal carcinoma of the left breast with distant metastases at the first presentation. The tumor was of high histological grade with negative estrogen receptors and positive c-erb B2 oncoprotein expression. This metastatic breast cancer did not respond to standard chemotherapy regimens including Trastuzumab, Taxanes and Doxorubicin.

MSI was present in 4 out of 8 loci in the tumor specimen. The IHC analysis revealed a negative expression for CK20, MSH2 and MSH6 and a positive expression for CK7 and MLH1 proteins, which confirm the mammary origin of the tumor and the role of the defective MSH2 gene in the pathogenesis (Tables 1 and 2).

**Table 1:** Microsatellite Instability (MSI) test results.

Locus	Stable	Unstable
BAT-25		×
BAT-26		×
D2S123/AFM093X43	×	
D17S250/MFd	×	
RIITGFβ	×	
NR21	×	
NR22		×
NR24		×

**Table 2:** Immunohistochemical (IHC) test results.

	Tumor tissue	Normal tissue
hMLH1	Positive	Positive
hMSH2	Negative	Positive
hMSH6	Negative	Positive
PMS2	Positive	Positive
CK7	Positive	
CK20	Negative	

MMR gene mutation carriers are at high risk of developing Lynch syndrome-related cancers. Some of them develop sporadic cancers due to exposure to non-genetic risk factors, polymorphisms in other genes and chance. These sporadic tumors may include breast cancer and usually exhibit stable microsatellite patterns and normal MMR protein expressions. The absence of an MSI-high pattern in a breast cancer specimen from MSH2 mutation carrier indicates that the development of such a tumor is unrelated to MMR gene impairment, despite the presence of the constitutional mutation. In contrast, a microsatellite instable early onset breast cancer that did not express the MSH2 protein in IHC and occurs in a proven MMR gene mutation carrier is highly suggestive of its belonging to the tumor spectrum of the disease [8,9]. Still, there is currently no evidence to suggest that, in general, female Lynch syndrome mutation carriers are at a strongly increased risk to develop breast cancer. In some cases, as we and others have shown, deficient DNA mismatch repair can contribute to breast cancer development in Lynch syndrome families. These findings do not necessarily imply that the underlying germline MMR mutation was also the strongest and most important risk factor in those breast cancer cases. Other genetic and non-genetic risk factors may have contributed and therefore all close relatives of breast cancer patients in Lynch syndrome families, including the relatives that do not carry the MMR mutation, might have an increased breast cancer risk. Testing for other germline mutation, e.g. of BRCA1, BRCA2 and possibly TP53, should be considered, especially in early-onset cases and unfortunately, we cannot exclude the presence of such mutation in our patient. It is not yet possible to accurately predict breast cancer risk for MMR mutation carriers in families with one or more cases of breast cancer. In lynch syndrome families; one might consider breast cancer surveillance for close female relatives of breast cancer cases, especially of those cases with early-onset breast cancer. Such surveillance might follow the guidelines for families with breast cancer and absence of detectable germline mutations in known hereditary breast cancer-associated genes. Although the efficacy of the surveillance program in reducing colorectal cancer mortality in Lynch syndrome has been proven, the benefit of breast cancer screening in selected Lynch syndrome families remains to be seen. In our opinion, cancers occurring in Lynch syndrome families which are not typical for that syndrome should undergo MSI analysis and immunostaining for the MMR gene protein expression in order to better assess the phenotype-genotype relationship and ultimately change the surveillance guidelines accordingly.
